# Stepwise inhibition of T cell recruitment at post-capillary venules by orally active desulfated heparins in inflammatory arthritis

**DOI:** 10.1371/journal.pone.0176110

**Published:** 2017-04-18

**Authors:** Hasan Al Faruque, Jin Hee Kang, Seung Rim Hwang, Shijin Sung, Md. Mahmudul Alam, Keum Hee Sa, Eon Jeong Nam, Young Ro Byun, Young Mo Kang

**Affiliations:** 1 Division of Rheumatology, Department of Internal Medicine, Kyungpook National University School of Medicine, Daegu, South Korea; 2 Department of Biochemistry and Cellular Biology, Kyungpook National University School of Medicine, Daegu, South Korea; 3 College of Pharmacy, Chosun University, Gwangju, South Korea; 4 Research Institute of Pharmaceutical Sciences, College of Pharmacy, Seoul National University, Seoul, South Korea; Macau University of Science and Technology, MACAO

## Abstract

Identification of the structure-function relationship of heparin, particularly between 2-O-, 6-O-, and N-sulfation and its anticoagulant or anti-inflammatory activities, is critical in order to evaluate the biological effects of heparin, especially in conjunction with modifications for oral formulation. In this study, we demonstrated that removal of 2-O, 6-O, or N-desulfation and their hydrophobic modifications have differential effects on the blocking of interactions between sLe^X^ and P-and L-selectins, with highest inhibition by 6-O desulfation, which was consistent with their *in vivo* therapeutic efficacies on CIA mice. The 6-O desulfation of lower molecular weight heparin (LMWH) retained the ability of LMWH to interfere with T cell adhesion via selectin-sLe^X^ interactions. Furthermore, 6DSHbD coated on the apical surface of inflamed endothelium directly blocked the adhesive interactions of circulating T cells, which was confirmed *in vivo* by suppressing T cell adhesion at post-capillary venular endothelium. Thus, in series with our previous study demonstrating inhibition of transendothelial migration, oral delivery of low anticoagulant LMWH to venular endothelium of inflamed joint tissues ameliorated arthritis by the stepwise inhibition of T cell recruitment and provides a rationale for the development of modified oral heparins as innovative agents for the treatment of chronic inflammatory arthritis.

## Introduction

T cells play a critical role in the pathogenesis of rheumatoid arthritis (RA) evidenced by the genetic association with major histocompatibility complex class II alleles and the T cell infiltrates within arthritic tissues [1, 2]. Recruitment of effector/memory T cells to specific sites of inflammation is an essential part of the immune response in chronic inflammatory disorders. As T cells contact activated endothelial cells at inflamed tissues, process of rolling, firm adhesion, and transendothelial migration occur through interaction between adhesion molecules, including selectins, intercellular adhesion molecules (ICAMs) and vascular cell adhesion molecules (VCAMs). Guidance cues, such as adhesion molecules and chemokines play a critical role in regulating T cell extravasation and infiltration in tissues during inflammatory response [3]. Thus, therapeutics that alter cell migration represent a particularly promising class of the new anti-inflammatory drug, such as anti-α4 integrin monoclonal antibody [4] and mimetics of sialyl-Lewis^X^ (sLe^X^) [5, 6].

Heparin is a highly sulfated, linear polysaccharide composed of alternating units of hexuronic acid and glucosamine [7]. In addition to its well-established anticoagulant activity, mediated by high-affinity binding to antithrombin via a unique pentasaccharide sequence, heparin has been proposed to play a regulatory role in limiting inflammation [8]. Indeed, therapeutic efficacy in clinical trials of patients with inflammatory disorders [9, 10] may support the potential anti-inflammatory effects of heparin.

Several mechanisms have been proposed to explain the anti-inflammatory activity of heparin [11]. The therapeutic effects of heparin have been mainly attributed to its ability to inhibit the interaction between leukocytes and activated endothelial cells (ECs) and to neutralize inflammatory mediators, such as chemokines and growth factors at the site of inflammation [12–14]. We have shown that 6-O desulfation of lower molecular weight heparin (LMWH) conjugated with deoxycholic acid inhibits transmigration of T cells through activated ECs and inhibits recruitment of T cells into inflamed arthritis tissues [15]. However, the efficacy of desulfation of other sites of LMWH on the anti-inflammatory functions has not been reported yet. Furthermore, regulatory mechanism of modified heparins on the direct interaction between ECs at post-capillary venules and T cells has not been fully elucidated.

The size and charge of heparin, however, are generally accepted to preclude absorption from the gastrointestinal tract, and make parenteral administration a necessity (NRI 2002). A variety of formulation and enhancing strategies to increase oral bioavailability of heparin have been investigated [16–19]. A lipidation strategy which involves conjugation of deoxycholic acid (DOCA) to facilitate its transport through the intestinal epithelium has been successfully applied to desulfated LMWH in our previous reports [15, 20]. Thus, identification of modified LMWHs that exert higher anti-inflammatory efficacy as well as lower anti-coagulant activity offers not only an insight into the mechanisms of heparin action, but also significant potential for development of oral agents. In this study, we investigated whether desulfated LMWHs at different sites of the disaccharide unit accompanied by conjugation with DOCA modulate the T cell adhesion on ECs during stepwise recruitment of T cells and whether they are differentially effective in suppressing disease activity in inflammatory arthritis.

## Materials and methods

### Reagents and antibodies

The sources of reagents are as follows: protease-free bovine serum albumin (BSA, Miles Inc., Kankakee, IL), polyacrylamide-sialyl Lewis^X^ (PAA-sLe^X^; Glycotech, Rockville, MD), carboxyfluorescein diacetate succinimidyl ester (CFSE; Molecular Probes, Eugene, OR), bovine type II collagen (CII), complete and incomplete Freund’s adjuvants (CFA and IFA, Chondrex, Redmond, WA), Bradford protein assay kit (Bio-Rad Laboratories, Hercules, CA), phytohemagglutinin (PHA; GibcoBRL, Rockville, MD), recombinant human IL-2 (Chiron, Amsterdam, NL), or mouse IL-2 (eBioscience, San Diego, CA). Recombinant human P-selectin-Fc chimera, TNF-α, and SDF-1α were purchased from R&D Systems (Minneapolis, MN). EDTA, Triton X-100, p-nitrophenyl N-acetyl-β-D-glucosaminide, Mayer’s H&E, protein A, and type IV collagenase were purchased from Sigma-Aldrich (Saint Luis, MO). Antibodies against human CD3ε (clone 14-2C11; eBioscience) and P-selectin (clone 9E1) were used.

### Heparin synthesis

**Fig 1:**
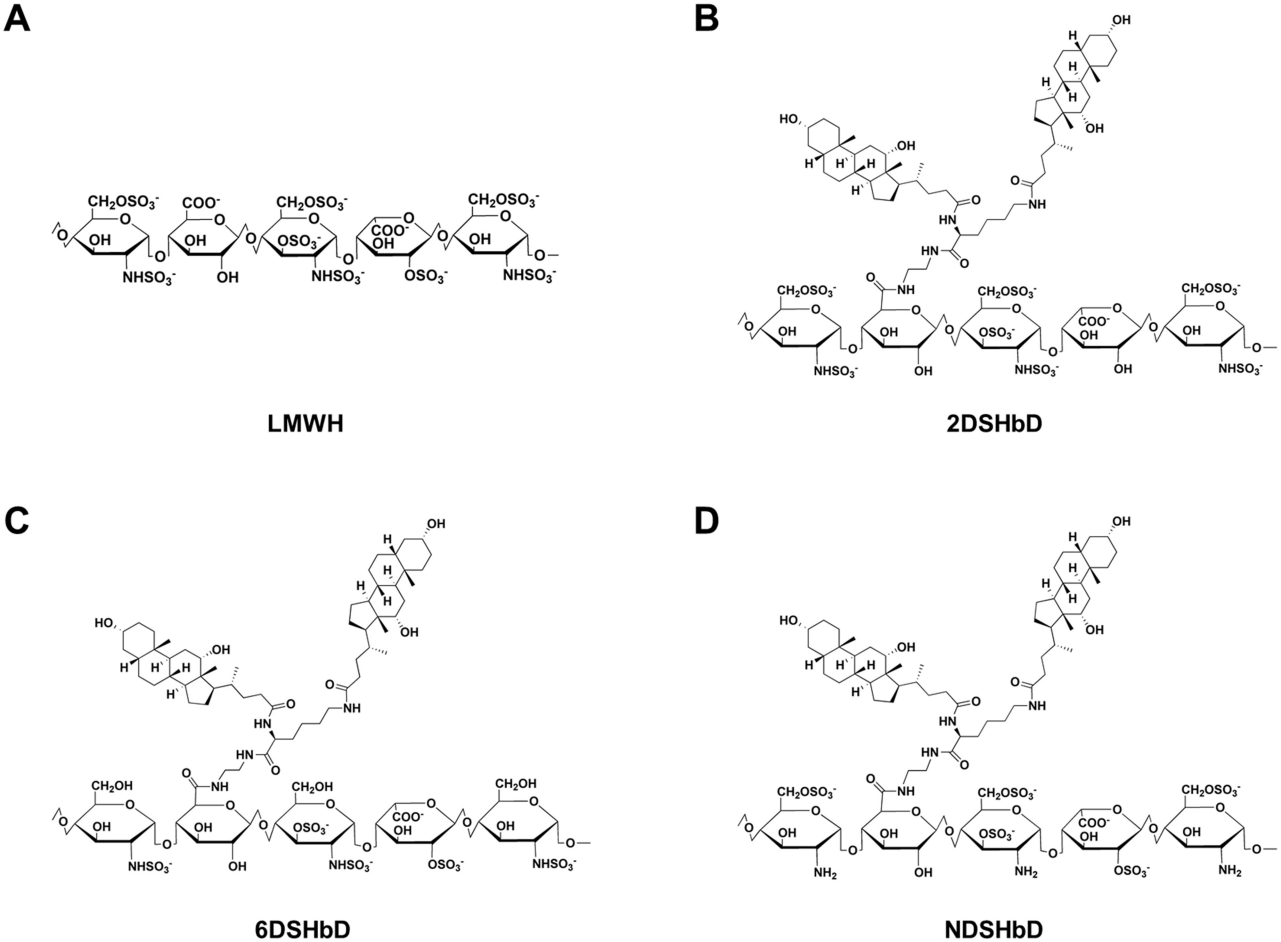
Structures of hydrophobically modified desulfated lower molecular weight heparins.

LMWH-*bis*DOCA (HbD), 2-O desulfated-HbD (2DSHbD), 6DSHbD and N-desulfated N-acetylated-HbD (NDSHbD) were synthesized as previously described [15, 20]. Briefly, N-deoxycholylethylamine (DOCA-NH2) or N-bisdeoxycholylethylamine (*bis*DOCA-NH2) was chemically conjugated to the carboxylic groups of LMWH, 2DS-LMWH, 6DS-LMWH and NDSHbD. To activate carboxylic groups, 1-ethyl-3-(3-dimethylaminopropyl) carbodiimide hydrochloride was added to dissolved LMWH, 2DS-LMWH, 6DS-LMWH, or NDSH-LMWH in formamide, DOCA-NH2 or bisDOCA-NH2 in formamide and/or dimethylformamide was added dropwise. The mixture was washed in cold ethanol after a 12 hours reaction. A Coatest anti-factor Xa (FXa) chromogenic assay (Chromogenix, Milano, Italy) was used to determine the anticoagulant activity. Sulfuric acid assay was used to determine the conjugation ratios of DOCA-NH2 or *bis*DOCA-NH2 to LMWH, 2DS-LMWH, 6DS-LMWH, or NDS-LMWH, respectively [21]. Structures of modified heparins were shown in Fig 1.

### ELISA inhibition assay

ELISA inhibition assays were performed as previously described [13]. Briefly, PAA-sLeX was coated (200 ng/well) onto ELISA plates (Costar, Cambridge, MA) by overnight incubation and then blocked with 1% protease-free BSA for 1 hour at 4°C. Heparin, buffer (positive control), or 10 mM NaEDTA (negative control) were preincubated with selectin-IgG-Fc chimera. Peroxidase-conjugated goat anti-human IgG (Jackson ImmunoResearch Laboratories) and the mixtures were added to coated wells. After incubation at 4°C for 4 hours, the plate was developed with O-phenylenediamine dihydrochloride. The absorbance was measured at 405 nm with a microplate reader (Molecular Device). The data were converted into percentages by using the formula: [(mean of duplicates)-(mean of negative control)]/[(mean of positive control)-(mean of negative control)] × 100.

### Cell culture

Human T cells were prepared by negative selection using magnetic bead separation (Miltenyi Biotec, Auburn, CA) following Ficoll-density gradient separation of peripheral blood mononuclear cells. This study was approved by the institutional review board of Kyungpook National University Hospital and written informed consent was obtained. Human umbilical vein endothelial cell (HUVECs, CRL-1730 ATCC) were cultured in EGM-2 medium and stimulated with TNF-α (10 ng/mL) for 16 hours before static and dynamic studies. Murine T cells were prepared by negative selection with magnetic separation kit (Miltenyi Biotec) from splenocytes followed by stimulation with PHA and murine IL-2 (20 ng/mL).

### T cell adhesion assay under static conditions

The T cell adhesion assay was performed as previously described with some minor modifications [22]. Briefly, recombinant human P-selectin-IgG-Fc chimera (0.1 μg/mL) or PAA-sLe^X^ (0.1 μg/mL) was coated onto ELISA plates overnight at 4°C. After blocking with 1% endotoxin-free BSA, T cells (2 × 10^5^/well) were incubated in coated wells containing heparin derivatives or EDTA (5 mM) for 2 hours. Adherent T cells were lysed using 1% Triton X-100 and p-nitrophenyl-N-acetyl-β-D-glucosaminide (3.75 mM) in citrate buffer. The absorbance was measured at 405 nm using a microplate reader.

### Analysis of lymphocyte motion under shear flow

Laminar flow adhesion assays were performed as described previously [23]. Immobilized P-selectin (1 μg/ml) bound to protein A (8 μg/ml) and TNF-α-stimulated confluent HUVECs were incubated on a 35-mm polystyrene dish. Effector T cells (1 × 10^6^/ml) preincubated with 6DSHbD or EDTA (5 mM) were perfused at 1.8 dynes/cm^2^ and recorded in high-power fields by videomicroscopy. Data were processed using MTrackJ (an ImageJ plug-in software created by Erik Meijering, Erasmus MC, Rotterdam, NL). Adherent cells were defined as cells that did not move during a 10 seconds interval, and interacting cells were defined as cells that interacted with the P-selectin or HUVEC monolayer for at least 1 second. Rolling flux and velocity were determined by counting the number of rolling cells and dividing the distance by the time within a field of view.

### Animal experiments

All animal care and experimental procedures were approved by the Kyungpook National University Institutional Animal Care and Use Committee (Approval Number: KNU 2012–35) and conducted in accordance with the institutional protocol for animal welfare. All studies involving animals are reported in accordance with the ARRIVE guidelines for reporting experiments involving animals [24, 25]. Male DBA/1J mice were obtained from SLC (Hamamatsu, Japan) and maintained under specific pathogen free conditions and a temperature-controlled environment with a 12/12 h light-dark cycle at the animal facility of Kyungpook National University School of Medicine. Standard lab chow and water were available *ad libitum*. All efforts were made for minimizing animal suffering. After completion of the study, mice were sacrificed with isoflurane and CO_2_ inhalation. Samples of the joints were collected for further analysis. Collagen-induced arthritis (CIA) was induced and scored according to a previously reported with minor modifications [26]. Briefly, DBA/1J mice between the ages of 6 and 8 weeks were immunized intradermally with 100μg of bovine CII in CFA or with IFA on day 0 and day 21. Mice were randomly assigned to the treatment groups upon the establishment of arthritis (n = 8 for each group). Clinical symptoms of arthritis were monitored three times per week from day 22 by two independent observers. Clinical arthritis index (CAI) was quantified by using a graded scale from 0 to 4 at the percipheral joints, as previously described [26]. *In vivo* anticoagulant activity was measured using prothrombin time (PT) and activated partial thromboplastin time (aPTT) after oral treatment with heparin derivatives (Chemon, Suwon, Korea) in CIA mice (control, n = 4; NDSHbD, n = 3; 2DSHbD, n = 3; 6DSHbD, n = 3).

### Semi-quantitative RT-PCR

The hind paws of randomly chosen CIA mice from each treatment group were homogenized in lysis reagent (Easy-spin; Intron Biotech, Sungnam, Korea). Total RNA was extracted using an RNeasy extraction kit (Intron Biotech) from the joint tissues after removing the skin of the hind paws. We employed 1 μg of total RNA for reverse transcription using oligo-dT and the PrimeScript 1st strand cDNA synthesis kit (Takara Bio, Shiga, Japan). Relative transcript levels for inflammatory mediators were quantified by real-time semiquantitative PCR, which was performed using primers, Taqman probes, and a LightCycler 480 system (Roche Diagnostics, Mannheim, Germany). The expression of inflammatory mediators was normalized to that of a target reference gene (18s ribosome RNA). The Taqman probes and oligonucleotides used are listed as follows: IL-1β: forward (F) (5′-TGTAATGAAAGACGGCACACC-3′), reverse (R) (5′-TCTTCTTTGGGTATTGCTTGG-3′), and probe (5′-CTGCTTCC-3′); IL-6: F (5′- GAGAAAAGAGTTGTGCAATGGC-3′), R (5′-CCAGTTTGGTAGCATCCATCA-3′), and probe (5′-TTCCCTCTG-3′); TNF-α: F (5′-CTGTAGCCCACGTCGTAGC-3′), R (5′-TTGAGATCCATGCCGTTG-3′), and probe (5′-ACGTCGTAG-3′); MMP-1: F (5′-TGTGTTTCACAACGGAGACC-3′), R (5′-GCCCAAGTTGTAGTAGTTTTCCA-3′), and probe (5′-CATCCAGG-3′); MMP-3: F (5′-TGTTCTTTGATGCAGTCAGC-3′), R (5′-GATTTGCGCCAAAAGTGC-3′), and probe (5′-GGGAGAAG-3′); VCAM-1: F (5′-TGGTGAAATGGAATCTGAACC-3′), R (5′-CCCAGATGGTGGTTTCCTT-3′), and probe (5′-AGGCAGAG-3′); RANKL: F (5′-TGAAGACACACTACCTGACTCCTG-3′), R (5′-CCACAATGTGTTGCAGTTCC-3′), and probe (5′-GGAGGATG-3′); MCP-1: F (5′-CATCCACGTGTTGGCTCA-3′), R (5′-GATCATCTTGCTGGTGAATGAGT-3′), and probe (5′-ACCTGCTG-3′); 18s ribosome RNA: F (5′-AAATCAGTTATGGTTCCTTTGGTC-3′), R (5′- GCTCTAGAATTACCACAGTTATCCAA-3′), and probe (5′-TCCTCTCC-3′).

### *In vivo* intra-vital microscopic analysis

Dorsal skin fold chambers (DSFC) were implanted on the back skin of CIA mice by surgical intervention according to previously described method with a minor modification [27, 28]. After chamber implantation, mice were kept in separate cages providing adequate amount of food and water. A recovery period of 7 days between implantation of DSFC and quantitative image analysis was allowed. CFSE labeled effector T cells (1 x 10^7^ cells) were suspended in PBS and 200 μl of suspension was injected in the tail vein of mice. Number of adherent T cells at the post-capillary venules was recorded with epifluorescence microscopy (OV-100, Olympus Co, Tokyo, Japan).

### Statistics

Statistical analysis was performed using SPSS version 12 (SPSS Inc., Chicago, IL). The difference between groups was analyzed by Student's t-test. For the comparison of the difference between treatment groups during multiple time points, repeated measures ANOVA with Tukey's post hoc test was performed. P value less than 0.05 was considered significant. Data are presented as the mean ± SEM unless otherwise indicated.

## Results

### Desulfations of LMWH conjugated with deoxycholic acid on the inhibition of selectin-sLe^X^ interaction

**Fig 2:**
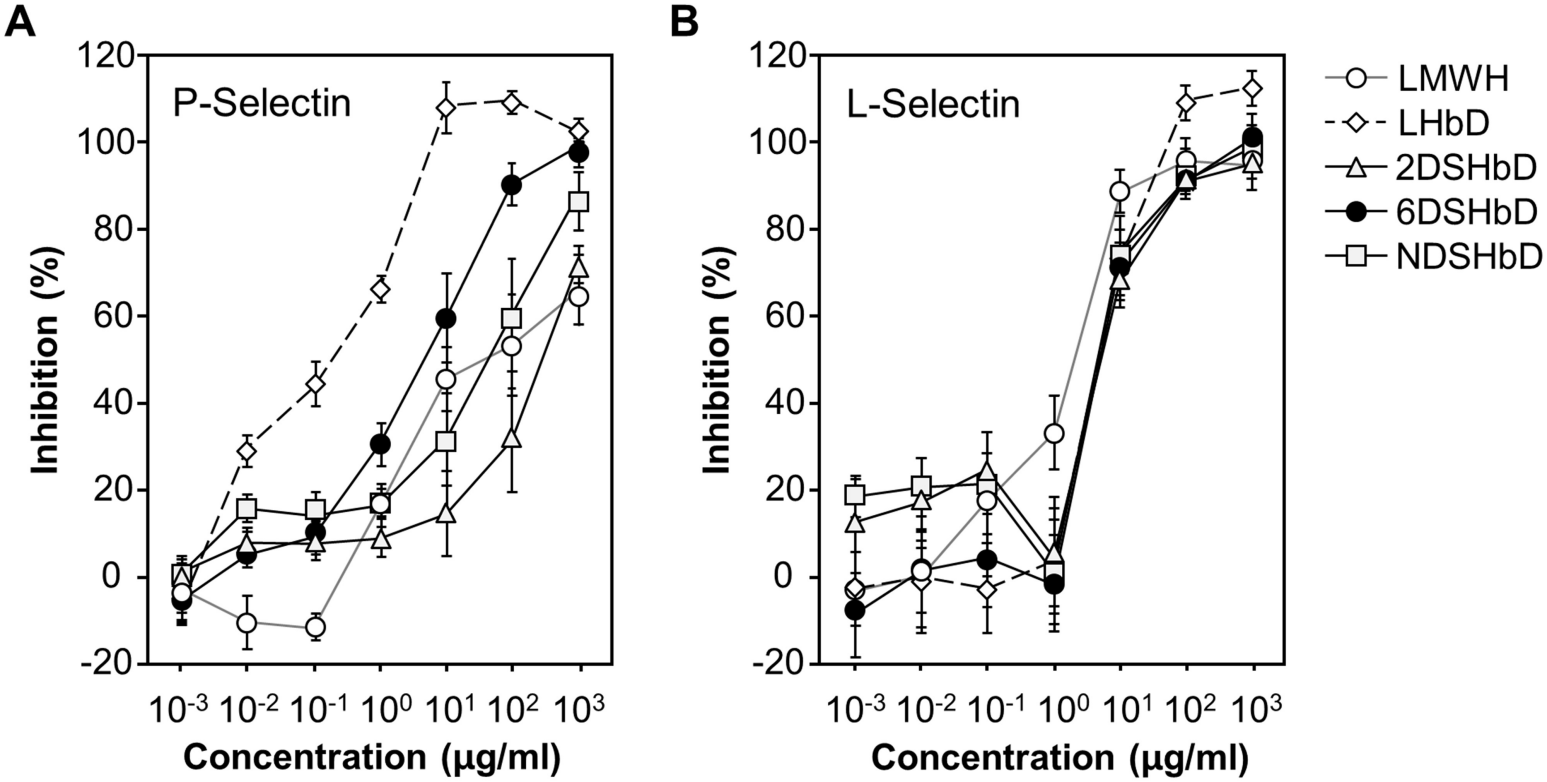
Inhibition of selectin-sLe^X^ interaction by desulfated heparins. Inhibition curves were produced by blocking with different concentrations of lower molecular weight heparin (LMWH, open circle), LMWH conjugated with *bis*DOCA (LHbD, open diamond), 2DSHbD (shaded triangle), 6DSHbD (closed circle), and NDSHbD (shaded square). Each point represents the mean ± SD of at least triplicate independent experiments.

To examine the antiadhesive role of modified LMWHs, we prepared regioselectively desulfated LMWHs and their conjugates with *bis*DOCA. The anti-FXa activity of 6DSHbD and NDSHbD was nearly completely abolished compared to that of LMWH, while 2DSHbD retained anti-FXa activity partially (7.1% relative to that of fraxiparine). We then tested their inhibitory efficacy on P-selectin-sialyl Lewis^X^ (sLe^X^) interaction (Fig 2A). Binding of P-selectin-IgG chimera to immobilized polyacrylamide-sLe^X^ was most efficiently inhibited by 6-O desulfated heparin compared with N-desulfated, 2-O desulfated, and non-desulfated LMWHs. IC_50_ value for inhibition of P-selectin-sLE^X^ interaction by 6DSHbD was 20.68 ± 6.75 μg/ml, while those of 2DSHbD, NDSHbD, and LMWH were 507.31 ± 29.50 μg/ml, 68.71 ± 5.20 μg/ml, and 101.67 ± 44.50 μg/ml. L-selectin-sLe^X^ binding, however, was inhibited to a similar extent by all these modified heparins (Fig 2B, IC_50_: 3.06 ± 0.29, 8.20 ± 0.70, 8.56 ± 0.85, and 8.76 ± 0.30 μg/ml for LMWH, 2DSHbD, 6DSHbD, and NDSHbD, respectively). These data showed that removal of 6-O, 2-O, and N-sulfations and their hydrophobic modifications with deoxycholic acid had differential effects on the inhibition of interactions between sLe^X^ and P- and L-selectins, thereby necessitating the comparison of *in vivo* anti-inflammatory effects of these materials in inflammatory arthritis models.

### Therapeutic efficacy of oral desulfated heparins in collagen-induced arthritis

**Fig 3:**
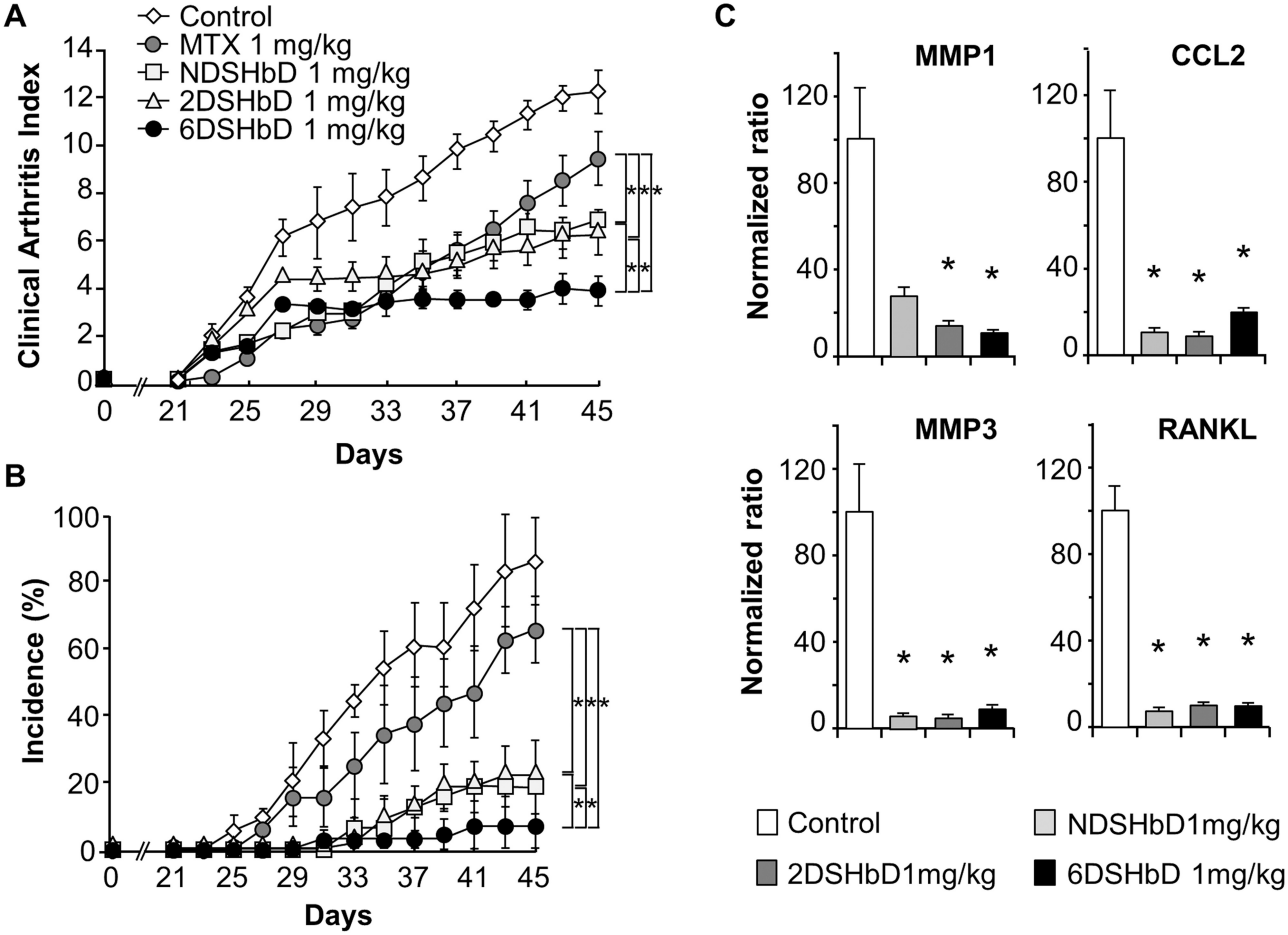
Therapeutic efficacy of oral desulfated heparins in murine collagen-induced arthritis. (A-B), CIA mice were treated with daily oral administration of 2DSHbD, 6DSHbD, and NDSHbD (1 mg/kg/day) or treated with a negative control (PBS, oral daily) and a positive control (Methotrexate 1mg/kg, two times/week, intraperitoneal). Efficacy of treatment was analyzed by CAI (A) and incidence of arthritic paws (B). Data represent the mean ± SEM (n = 8 for each group). *p < 0.05. (C), Transcript level of inflammatory mediators in the joint tissues from control and desulfated heparin-treated CIA mice. Semiquantitative real-time PCR was performed using Taqman probes and analyzed using a LightCycler480 system. Data represent the mean ± SEM. *p < 0.05 versus control.

We next determined whether *bis*DOCA conjugated LMWHs with distinct desulfations have differential efficacy on the inflammation using murine CIA model which has T cell-mediated pathophysiology [29, 30]. In this murine model, joint symptoms became apparent between days 21 and 23 and peak between days 40 and 45 after the first immunization. We treated mice with CII to induced CIA and then administered modified heparins via gavage beginning from day 23 after the first immunization for the following days until day 45 (Fig 3). Anticoagulant activities measured by PT and aPTT revealed no significant different between the control group and modified heparin treated groups (Table 1).

**Table 1:** Anticoagulant activities after oral treatment with heparin derivatives in collagen-induced arthritis mice.

Heparin derivatives	Anticoagulation activities	
	PT (sec)	aPTT (sec)
Control (n = 4)	7.18 ± 0.41	20.30 ± 1.36
NDSHbD (n = 3)	7.47 ± 0.12	20.23 ± 0.76
2DSHbD (n = 3)	7.53 ± 0.15	19.97 ± 0.49
6DSHbD (n = 3)	7.30 ± 0.20	19.03 ± 1.29

NDSHbD, N desulfated LMWH conjugated with *bis*-DOCA; 2DSHbD, 2-*O* desulfated LMWH conjugated with *bis*-DOCA; 6DSHbD, 6-*O* desulfated LMWH conjugated with *bis*-DOCA. LMWH, Low molecular weight heparin; DOCA, deoxycholic acid; PT, Prothrombin time; aPTT, Activated partial thromboplastin time.

Upon measurement of CAI and incidence of arthritis on the paws, mice treated with all three modified heparins (1mg/kg/day, p.o.) showed significantly reduced severity of arthritis compared with methotrexate (1 mg/kg, two times a week, i.p.) and control (PBS, daily, p.o.). Among these modified heparins, 6DSHbD ameliorated the arthritis more effectively compared to 2DSHbD and NDSHbD (Fig 3A and 3B, *p* < 0.05).

To characterize effects of modified heparins on the inflammatory response within joint tissues, we quantified transcripts of inflammatory mediators from the whole extracts of arthritic tissues. Suppression of inflammation, as evidenced by reduced inflammatory transcripts within joints, was found in CIA mice treated with all three modified heparins compared to controls (Fig 3C). These data showed that 6DSHbD was most effective in ameliorating severity of CIA after oral administration as well as it retained the least anticoagulant activity among different desulfated LMWHs. Thus, we chose 6DSHbD for subsequent experiments in this study.

### Inhibition of selectin-mediated T cell adhesion by 6DSHbD in static and dynamic conditions

**Fig 4:**
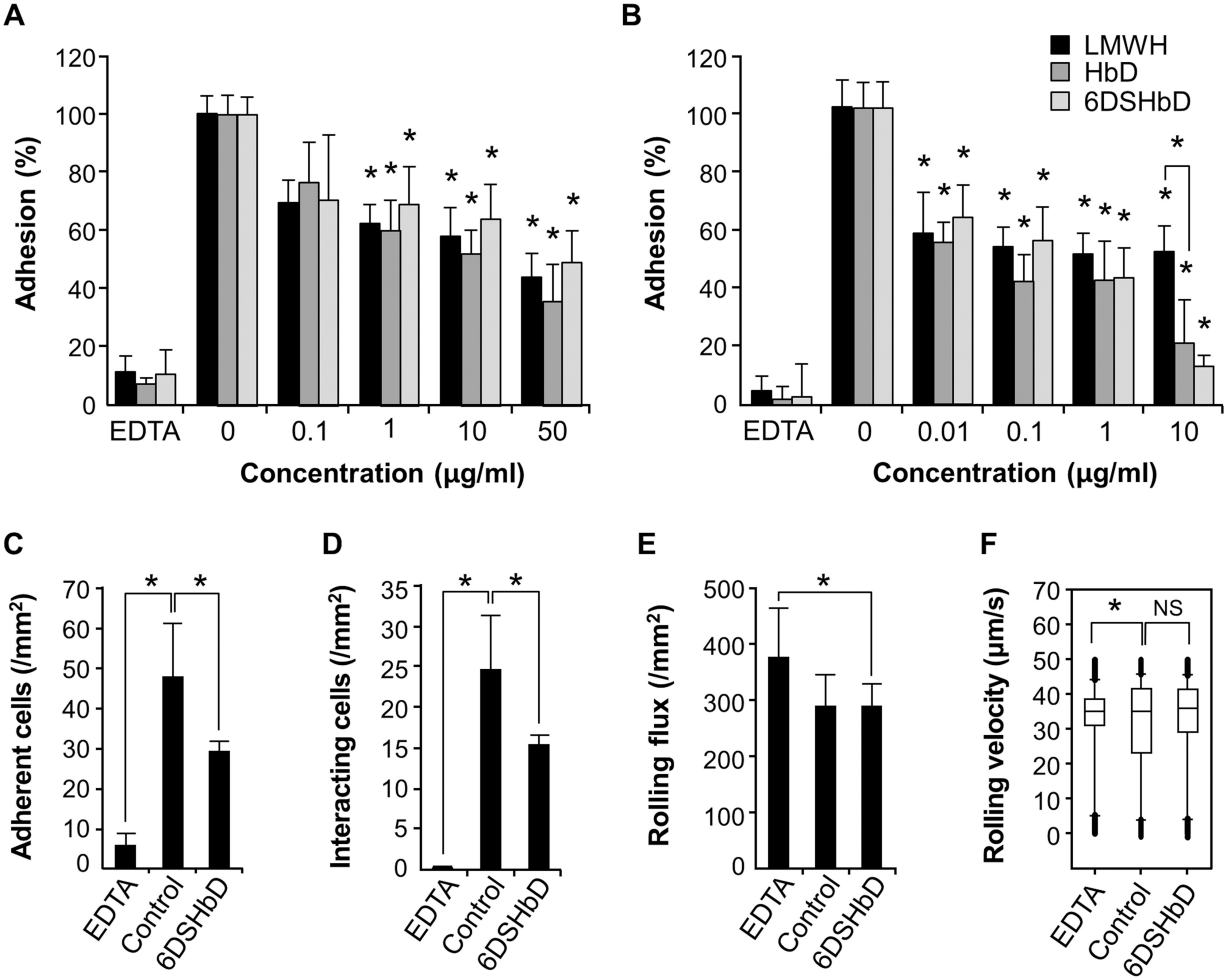
Inhibition of selectin-mediated T cell adhesion by 6-O desulfation of LMWH (6DSHbD) in static and dynamic conditions. (A-B), Inhibition of adhesion of T cells on the (A) P-selectin-IgG-Fc chimera- and (B) PAA-sLe^X^-coated plates under a static condition. Data represent the mean ± SEM from 3 independent experiments. *p < 0.05 versus no treatment. (C-F), Interaction of T cells with P-selectin under flow. P-selectin bound to protein A on a 35-mm polystyrene dish was pre-incubated with or without 6DSHbD. T cells (1 × 10^6^/ml) were pre-incubated with 5 mM EDTA buffer or 6DSHbD(100 μg/ml) and placed under flow at a wall shear stress of 1.8 dynes/cm^2^ over the P-selectin-coated dish. The number of adherent (C), interacting (C), or rolling (D) T cells was counted in 5 random high-power fields for each condition at 10 seconds intervals and expressed as cells/mm^2^. (E) The rolling velocity of T cells was measured and expressed as μm/s. Data represent the mean ± SEM from 3 independent experiments. *p < 0.0.5

P-selectin glycoprotein ligand-1 (PSGL-1) plays a central role in the trafficking of lymphocytes to areas of inflammation by direct interaction with P- and L-selectins via sLe^X^ and a sulfate group [31, 32]. To determine whether 6DSHbD modulates the selectin-mediated adhesive processes, we tested the adhesion of T cells to either P-selectin-IgG chimera or PAA-sLe^X^ immobilized on plastic plates. In contrast to the results on the selectin-sLe^X^ interaction, the adhesion of T cells to P-selectin was inhibited by 6DSHbD to a similar extent compared with LMWH (Fig 4A). Furthermore, the inhibition of sLe^X^-mediated T cell adhesion, which indicates sLe^X^-L-selectin interaction, was greater by 6DSHbD than by LMWH (IC_50_: 6.26 ± 3.05 μg/mL vs. 47.93 ± 20.79 μg/mL for 6DSHbD vs. LMWH, respectively, *p* < 0.05) (Fig 4B).

To more closely approach the physiological situation, we used phase-contrast videomicroscopy to study the effect of modified heparins on the adherence of T cells to immobilized P-selectin under physiological laminar shear flow. Approximately 47.8 ± 13.5 cells/mm^2^ contacting P-selectin bound to protein A adhered to the plate surface. In comparison, 29.1 ± 2.8 cells/mm^2^ treated with 6DSHbD adhered to P selectin-coated surface (Fig 4C, *p* < 0.05). The number of interacting T cells also was significantly reduced by treatment with 6DSHbD compared to control (Fig 4D). However, rolling flux and velocity were not influenced by 6DSHbD when compared to control (Fig 4E and 4F). These findings showed that 6-O-desulfation of LMWH retained the ability of LMWH to interfere with the adhesion between T cells and endothelial cells via selectin–sLe^X^ interactions in both static and dynamic conditions.

### Inhibition of T cell adhesion on activated endothelial cells by 6DSHbD in static and dynamic conditions

The early steps that promote recruitment of effector/memory T cells to non-lymphoid tissues and sites of inflammation include selectin-mediated rolling, chemokine-triggered activation, and integrin-dependent arrest on the endothelial surface [32]. We have shown that 6DSHbD displayed greater binding to TNF-α stimulated HUVECs compared to unstimulated cells, followed by internalization into ECs [15]. We examined whether surface-bound modified heparin can directly block the adhesion of T cells to HUVEC monolayers under both static and dynamic conditions.

**Fig 5:**
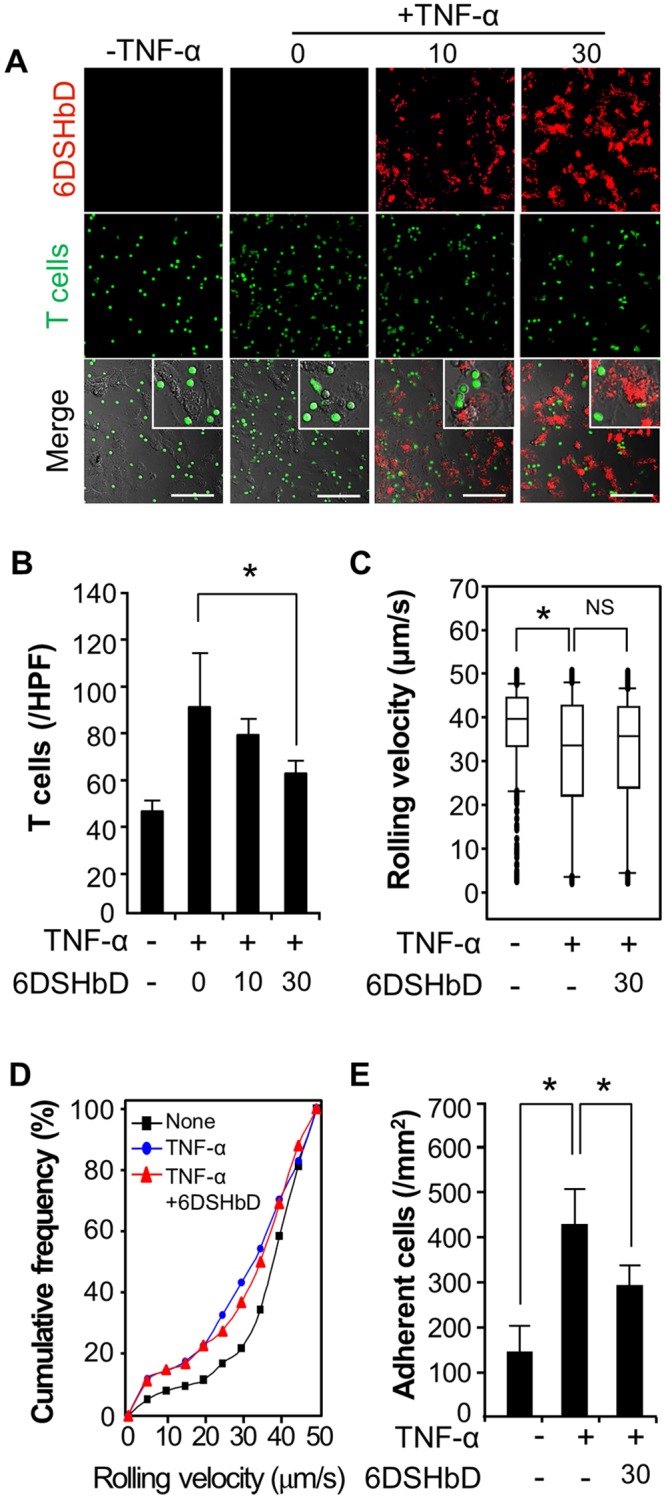
Inhibition of T cell adhesion on activated ECs by 6DSHbD in static and dynamic conditions. (A–B), Unstimulated or TNF-α-stimulated HUVEC monolayers were incubated with 6DSHbD-Cy5.5 (μg/ml, red) for 30 minutes and co-cultured with CFSE-labeled T cells (green) in a static condition. Fluorescence microscopy of the adherent T cells with HUVECs (A) and the number of adherent T cells was counted in 5 random high-power fields (B). Scale bars, 100 μm. (C-E), Unstimulated or TNF-α-stimulated HUVEC monolayers were treated with 6DSHbD and placed in a parallel plate-flow chamber device. T cells were allowed to interact with HUVEC monolayers at a shear stress of 1.8 dynes/cm^2^. The rolling velocity of T cells (C), cumulative frequency of the rolling velocities of T cells (D), and the number of adherent T cells on HUVECs (E) were analyzed.

Under static conditions, 6DSHbD-Cy5.5 (red) bound on the surface of TNF-α-stimulated HUVECs in a dose-dependent manner. The adhesion of T cells labeled with CFSE (green) on the HUVECs was excluded from the surface of endothelial cells where 6DSHbD-Cy5.5 was accumulated (Fig 5A). The number of T cells adherent to the surface of HUVECs decreased significantly following treatment with a higher concentration (30 μg/ml) of 6DSHbD (Fig 5B).

The efficiency of T cell recruitment, as determined by the conversion from rolling to arrest in post-capillary venules, was recapitulated on TNF-α-stimulated HUVECs in the parallel-plate flow chamber [23]. Rolling and adhesive interactions of T cells with HUVEC monolayers were examined under defined laminar flow. The average rolling velocity of T cells on activated HUVECs under a flow of 1.8 dynes/cm^2^ was significantly lower compared with that on unstimulated HUVECs. After treatment with 6DSHbD, both the average rolling velocity and cumulative frequency over the rolling velocity of T cells were slightly elevated compared to those of untreated cells, although the differences were not statistically significant (Fig 5C and 5D). The number of stably arrested T cells on stimulated endothelial cells was significantly decreased by 6DSHbD treatment (426 ± 82 vs. 291 ± 44 cells/mm^2^, *p* < 0.05) (Fig 5E). These findings show that 6DSHbD coated on the apical surface of inflamed endothelium directly interferes with the adhesive interactions of circulating T cells with endothelial cells.

### Reduced T cell adhesion on the activated venular endothelium under the intra-vital microscopy

**Fig 6:**
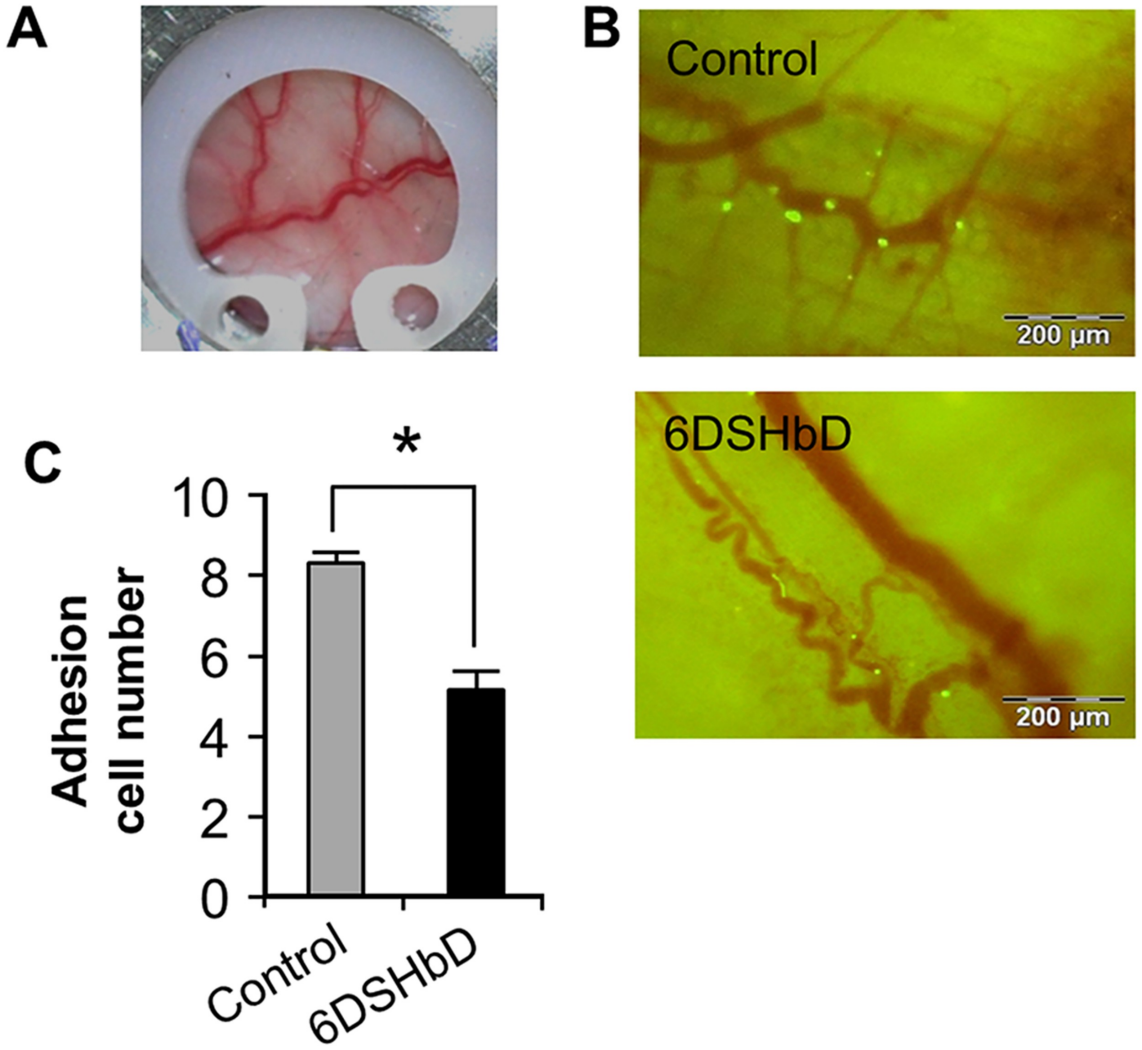
Reduced T cell adhesion to the post-capillary venular endothelium on intravital microscopy. (A) Image of implanted dorsal skinfold chamber on back of DBA/1J mouse. (B) Representative images of intravital microscopy of CFSE-labeled T cell adhesion on endothelium at post-capillary venules in vivo. (C) Quantification of adherent T cells on the endothelial surface in control (n = 3) and 6DSHbD treated mice (n = 3). Data are mean ± SEM from 3 independent experiments. *p < 0.05.

To evaluate the efficacy of oral 6DSHbD on the diapedesis of T cells through the post-capillary venules *in vivo*, we prepared a modified DSFC using small round chamber covered with a removable glass and implanted this chamber on the back skin of DBA1/J mice. Mice were treated orally with 6DSHbD (10 mg/kg) for 3 days, followed by intravenous injection of CFSE-labeled effector T cells (2 x 10^6^ cells). Prior to T cell transfer, cover glass was removed and the exposed vessels were stimulated with TNF-α (10 ng/ml) and a chemokine (SDF-1α, 10 μg/ml) for 4 hours (Fig 6A). Venules with diameter between 40 and 80 μm were selected and the adherent and transmigrating T cells (green) were recorded using fluorescent videomicroscopy (Fig 6B). The number of adherent T cells on the venular surface was significantly reduced in 6DSHbD treated mice (Fig 6C). These findings confirmed that 6DSHbD, accumulated on the activated endothelium, directly inhibited the adhesion of effector T cells on the endothelial cell surface at the post-capillary venules *in vivo*.

## Discussion

Identification of the structure-function relationship of heparin, particularly between 2-O-, 6-O-, and N-sulfation and its anticoagulant or anti-inflammatory activities, is critical in order to evaluate the biological effects of heparin, especially in conjunction with modifications for oral formulation [8, 13]. In the present study, we demonstrated that removal of 2-O, 6-O, and N-desulfations and their hydrophobic modifications have differential effects on the blocking of interactions between sLe^X^ and P-and L-selectins, with highest inhibition by 6-O desulfation, which was consistent with *in vivo* therapeutic efficacy on CIA mice. The 6-O desulfation of LMWH retained the ability of LMWH to interfere with T cell adhesion via selectin-sLe^X^ interactions. Furthermore, 6DSHbD coated on the apical surface of inflamed endothelium directly blocked the adhesive interactions of circulating T cells with activated endothelial cells, which was confirmed *in vivo* by suppressing T cell adhesion at post-capillary venules.

The 2-O- and 3-O-sulfation provide favorable contacts between heparin and antithrombin, and 6-O-sulfate group appears to grant increased catalytic efficacy to anti-thrombin [33]. We observed that 6-O-desulfation and N-desulfation of LMWH abrogated anticoagulant activity, whereas 2-O-desulfation produced only a partial reduction in anticoagulant activity, which is consistent with studies on the anticoagulant efficacy of regioselectively desulfated natural heparins [13, 34]. Removal of the anticoagulant function of LMWH, however, did not affect its ability to block T cell-endothelial cell adhesion via selectin-sLe^X^ interactions. Whereas the 6-O-sulfate of glucosamine is important for binding to selectin [13] and FGF2 [35], 2-O- and/or 3-O-desulfation of unfractionated heparin inhibits the adhesion of monocytes to selectins [13] or the receptor for advanced glycation endproducts (RAGE) [36], as well as the adhesion of neutrophils to endothelial cells [34]. Partial 6-O-desulfation may retain enough anionic charge to mediate electrostatic interactions with sufficient affinity to preserve the biologic properties of heparin [37].

In murine CIA model, all three desulfated heparins were more effective for the treatment of arthritis compared to methotrexate, which is a standard disease modifying anti-rheumatic drug. Among desulfated heparins, 6DSHbD showed highest therapeutic efficacy. These data suggest an effect of 6-O desulfation on anti-inflammatory effects which could be direct and/or indirect. Data in Fig 2 argue for a direct effect by more effective blocking of P-selectin-sLe^X^ binding. Previous studies revealed higher bioavailability of 6DSHbD compared to 2DSHbD. Furthermore, intracellular accumulation of hydrophobically modified desulfated heparins may result in the differential regulation on signaling pathways that require further investigation [15].

Unfractionated heparin binds to and is internalized by endothelial and vascular smooth muscle cells and is involved in the regulation of signaling pathways [38, 39]. Heparin is cleared by both rapid saturable and slower, first-order mechanisms. The rapid saturable clearance phase following intravenous injection may be due to the binding of heparin to endothelial cells and its subsequent uptake [40]. In contrast, LMWH displays reduced binding to HUVECs, which may result in its clearance by primarily nonsaturable or renal mechanisms [40]. We have previously shown that 6DSHbD internalized into endothelial cells after binding to P-Selectin and VCAM-1 which leads to the inhibition of transcellular migration of T cells by blocking RhoA GTPase activation [15]. In the present study, we further demonstrated that 6DSHbD bound on the surface of activated ECs directly hampered the adhesion of T cells on the ECs, thus reducing the chance to be recruited into inflamed joint tissues prior to internalization of 6DSHbD.

An essential step in this process is the adhesion of effector T cells to the endothelium of post-capillary venules—a complex multistep cascade of events mediated by adhesion receptors [41], such as P-selectin and VCAM-1, which varies depending on the target tissue and the inflammatory context. Inhibition of T cell homing to synovial tissue (ST) represents a potential strategy for the treatment of chronic inflammatory arthritis. Another critical step, we have shown, was that the oral administration of 6DSHbD resulted in its preferential accumulation within inflamed ST, particularly around post-capillary venules [15]. Moreover, reduced homing of effector T cells to inflamed ST that occurred in response to the oral 6DSHbD was dependent on P-selectin-mediated cellular accumulation of 6DSHbD [15]. However, there has been mechanistic gap between the accumulation of 6DSHbD and reduced homing of effector T cells, which may be explained either by the direct inhibition of T cell adhesion on ECs or by sequestration of chemokines [42, 43]. Actually, neutralization of multiple CC chemokines, which may also achieved by heparin especially via inhibition of oligomerization [44], resulted in a reduction of disease activity in an arthritis model through the retention of effector T cells [43, 45]. The results of the present study may fill this gap by clearly demonstrating that 6DSHbD directly inhibited adhesion of T cells on the endothelium of post-capillary venules by blocking the tethering and adhesion of T cells on 6DSHbD-coated surface of endothelium.

To our knowledge, this is the first report to show that oral delivery of low anticoagulant LMWH to venular endothelium of inflamed joint tissues protects from arthritis. This effect is mediated by the stepwise inhibition of T cell recruitment and provides a rationale for the development of modified oral heparins as innovative agents for the prevention and/or treatment of chronic inflammatory arthritis.

## Abbreviations

2DSHbD: 2-O desulfated-LMWH-*bis*deoxycholic acid
6DSHbD: 6-O desulfated-LMWH-*bis*deoxycholic acid
CAI: clinical arthritis index
CFSE: Carboxyfluorescein succinimidyl ester
CIA: collagen-induced arthritis
DOCA: deoxycholic acid
DSFC: dorsal skinfold chamber
HbD: LMWH-*bis*deoxycholic acid
HUVEC: human umbilical vein endothelial cell
ICAM: intercellular adhesion molecule
IL-2: interleukin-2
LMWH: lower molecular weight heparin
NDSHbD: N-desulfated N-acetylated-LMWH-*bis*deoxycholic acid
RA: rheumatoid arthritis
SDF-1α: stromal cell-derived factor-1α
sLe^X^: sialyl-Lewis^X^
TNF-α: tumor necrosis factor-α
VCAM: vascular cell adhesion molecule
